# Development and validation of a nomogram model for prediction of dyslipidemia in children with Wilson disease: a retrospective analysis

**DOI:** 10.3389/fendo.2025.1642083

**Published:** 2025-08-21

**Authors:** Daiping Hua, Qiaoyu Xuan, Lanting Sun, Wei Song, Wenming Yang, Han Wang

**Affiliations:** ^1^ Department of Neurology, The First Affiliated Hospital of Anhui University of Chinese Medicine, Hefei, China; ^2^ Information Center, The First Affiliated Hospital of Anhui University of Chinese Medicine, Hefei, China; ^3^ Key Laboratory of Xin’an Medicine, Ministry of Education, Hefei, China

**Keywords:** Wilson disease, children, dyslipidemia, nomogram, prediction, risk factors

## Abstract

**Background:**

Wilson disease (WD), an inherited copper metabolism disorder, is linked to hepatic injury from copper accumulation-induced dyslipidemia. Children with WD have a high incidence of dyslipidemia, yet personalized risk assessment tools are lacking. This study established a predictive nomogram to provide foundational evidence for early detection in this population.

**Methods:**

In this retrospective cohort study, clinical data from 913 children with WD were retrospectively collected at the First Affiliated Hospital of Anhui University of Chinese Medicine (November 2018–February 2025). The cohort was stratified by age group and dyslipidemic status using stratified random sampling, resulting in a division into a training set (70%, *n* = 641) and a validation set (30%, *n* = 272). Independent risk factors were identified using least absolute shrinkage and selection operator (LASSO) regression and multivariate logistic regression analyses. The nomogram prediction model was constructed and validated internally. The model’s discriminatory efficacy was evaluated using Receiver Operating Characteristic (ROC) curves with the area under the curve (AUC), while its calibration performance was assessed using calibration curves and the Hosmer-Lemeshow test. Furthermore, the clinical utility of the model was examined through decision curve analysis and clinical impact curves.

**Results:**

The prevalence of dyslipidemia was 68.24%. The nomogram incorporated six significant clinical variables: age group (≥ 10 years vs. < 10 years), alanine aminotransferase (ALT), gamma-glutamyl transpeptidase (GGT), homocysteine (Hcy), superoxide dismutase (SOD), and platelet count (PLT). The prediction model demonstrated good discrimination (AUC: 0.810 in the training set, 0.831 in the validation set), excellent calibration (Hosmer-Lemeshow *P* > 0.280), and significant clinical utility.

**Conclusion:**

Children with WD exhibit a high incidence of dyslipidemia. The nomogram prediction model based on these six variables effectively predicts dyslipidemic risk in pediatric WD patients, enabling early identification and clinical risk stratification.

## 1 Introduction

Wilson disease (WD) is an inherited disorder of copper metabolism caused by mutations in the ATP7B gene, leading to pathological copper deposition in the liver and other tissues (1). There is a strong link between copper and lipids (2, 3), excessive copper deposition in the liver leads to dyslipidemia, and the accumulation of lipid droplets in hepatocytes induces hepatic steatosis via various pathways, such as oxidative stress and lipid peroxidation, which in turn leads to hepatic inflammation, necrosis, fibrosis, and even cirrhosis (4–6).

According to our previous studies by Chinese scholars, the prevalence of WD in China is approximately 5.87/100,000 (7), which is higher than that reported in Western countries. Children and adolescents account for the majority of WD cases (8), and approximately 80% of pediatric WD patients are more likely to present with liver damage than adults are (9). Previous studies revealed that the incidence of dyslipidemia in children with WD is high and that the risk of liver fibrosis is increased (10). Studies indicate that childhood risk factors track to adulthood and predict surrogate end points (11). There have been few reports of dyslipidemic risk factors in children with WD, so early identification and intervention of dyslipidemia in children with WD are highly important for slowing the progression of the disease.

This retrospective study was conducted on patients with WD who received treatment at the First Affiliated Hospital of Anhui University of Chinese Medicine. This institution is recognized as one of the largest and most authoritative international centers for the diagnosis and treatment of WD, having managed 40,000 patients from 15 different countries and regions. Furthermore, our preliminary research group has established the world’s most extensive International Wilson Disease Mutation Database (accessible at https://wilsondisease.azyfy.com/), which compiles nearly all *ATP7B* mutations documented in the scientific literature up to August 2024. The database currently encompasses a total of 1,610 identified mutations. To establish temporal precedence for risk assessment, we employed a retrospective cohort design wherein dyslipidemia outcomes were longitudinally confirmed per standardized diagnostic criteria. Therefore, the aim of this study was to investigate the prevalence of dyslipidemia in children with WD, explore independent risk factors for dyslipidemia in pediatric WD, and develop a validated nomogram for risk stratification. This model integrates readily accessible and easily detectable biomarkers to offer clinicians an intuitive and user-friendly tool for the rapid assessment of dyslipidemia risk in children with WD. This approach is particularly advantageous in resource-limited healthcare settings. This study provides guidance for clinicians to recognize dyslipidemia in children with WD at an early stage, thus delaying the process of liver damage in children with WD.

## 2 Materials and methods

### 2.1 Participants

**Figure 1:**
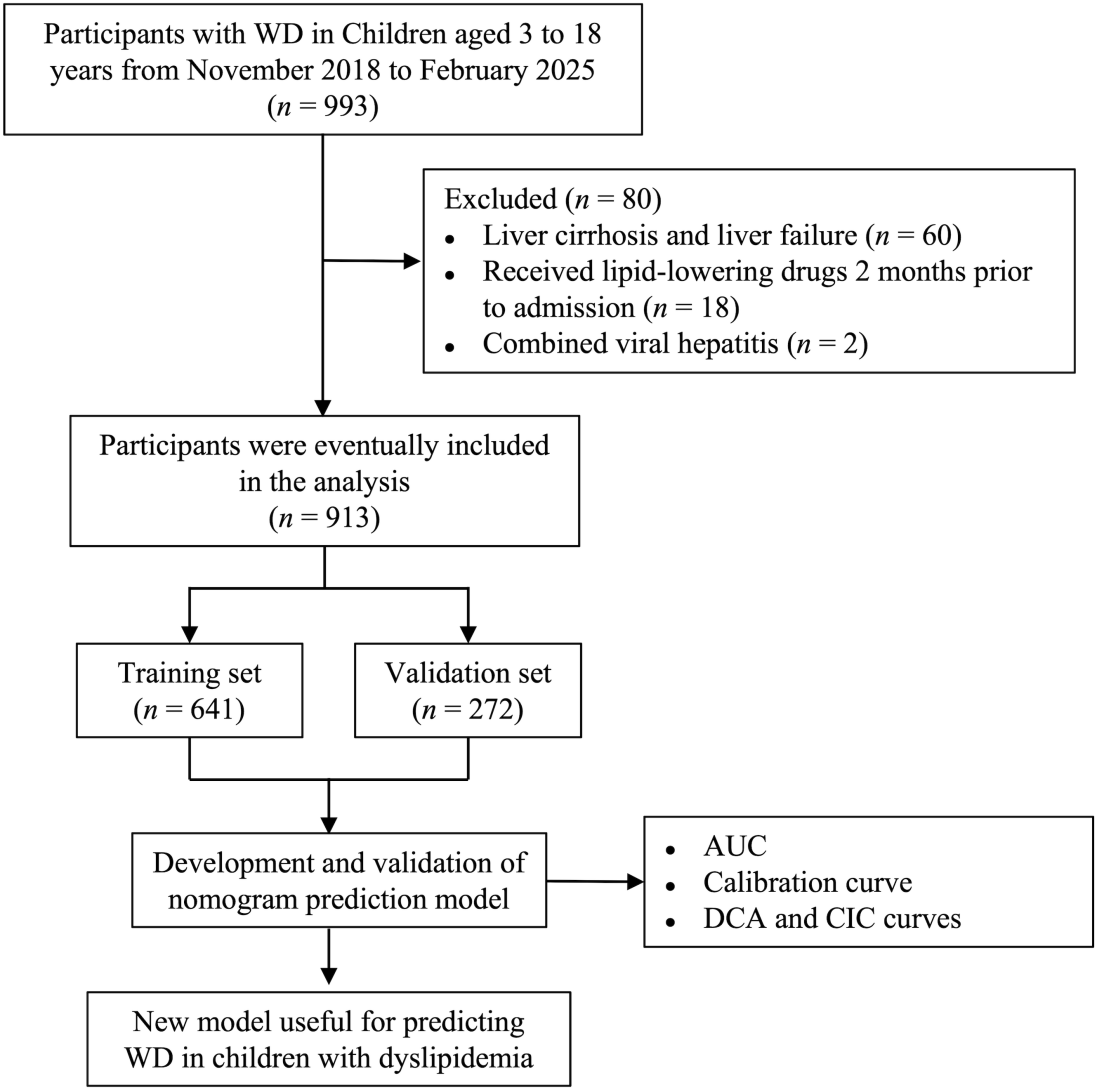
Flowchart of the study participants.

The data of 993 children with WD who were first admitted to the First Affiliated Hospital of Anhui University of Chinese Medicine from November 2018 to February 2025 were included (**Figure 1**). The inclusion criteria were as follows: (1) newly diagnosed WD meeting the diagnostic criteria for WD and (2) aged 3 to 18 years. The exclusion criteria were as follows: (1) had abnormal liver function caused by autoimmune hepatitis, viral hepatitis or other diseases; (2) had liver cirrhosis or liver failure; and (3) were taking lipid-lowering drugs before admission (2 months). All biomarker measurements represent pre-treatment status, with anti-copper therapy commencing after initial assessment. The 913 children with WD were randomized into the model population and the validation population at a ratio of 7:3. The protocol was approved by the Medical Ethics Committee of the First Affiliated Hospital of Anhui University of Chinese Medicine with approval number 2023MCZQ08.

### 2.2 Diagnostic criteria

#### 2.2.1 Diagnostic criteria for WD

According to the Leipzig scoring system (12), a total score of ≥ 4 was sufficient to confirm the diagnosis of WD.

#### 2.2.2 Diagnostic criteria for dyslipidemia

In accordance with the diagnostic thresholds issued by the National Cholesterol Education Program, as recommended by the expert consensus on the diagnosis and management of dyslipidemia in children (13–15), dyslipidemia can be classified into hypercholesterolemia, hypertriglyceridemia, mixed hyperlipidemia, hyper lipoprotein(a)emia, and low high-density lipoprotein hyperlipidemia. The diagnostic reference values were as follows: total cholesterol (TC) ≥ 5.17 mmol/L, triglyceride ≥ 1.12 mmol/L at <10 years of age, triglyceride ≥ 1.46 mmol/L at ≥ 10 years of age, lipoprotein(a) ≥ 300 mg/L (16), and high-density lipoprotein-cholesterol (HDL-C) < 1.03 mmol/L. Dyslipidemia was characterized by two consecutive fasting measurements of lipid components that exceeded or fell below the specified thresholds. The normolipidemic group and dyslipidemic group were categorized according to the occurrence of dyslipidemia.

### 2.3 Data collection

The following data were extracted from the hospital information system (HIS): (1) Basic information: sex, age, and body mass index (BMI). (2) Biochemical indicators: TC, triglyceride, HDL-C, low-density lipoprotein cholesterol (LDL-C), apolipoprotein A-I (ApoA-I), apolipoprotein B (ApoB), the ApoA-I/ApoB ratio, lipoprotein(a), alanine aminotransferase (ALT), aspartate transaminase (AST), gamma-glutamyl transpeptidase (GGT), albumin, total bilirubin (TBIL), total bile acids (TBA), homocysteine (Hcy), superoxide dismutase (SOD), platelet count (PLT), fibrinogen, uric acid (UA), serum creatinine, and blood urea nitrogen (BUN); (3) Copper biochemical indicators: serum ceruloplasmin, 24-h urine copper, and serum copper.

### 2.4 Statistical analysis

Count data were expressed as numbers (percentages), and compared using the *χ^2^* test; continuous variables were described as mean ± standard deviation (normally distributed) or median (interquartile range, IQR; nonnormally distributed) with group comparisons via Student’s *t* test or *Mann–Whitney U* test, respectively. Missing data (*n* = 29) were handled *via* multiple imputation (5 datasets, 5 iterations) using the “mice” package, with pooled results calculated using Rubin’s rules (17). The proportion of missing data for variables used in all regression analyses is described in **Supplementary Table S1**. To ensure reproducibility, a fixed random seed (1357) was set before splitting the dataset, which was divided using stratified sampling based on dyslipidemic status and age group. For model development, 913 participants were randomly split into training (*n* = 641) and validation (*n* = 272) groups in a 7:3 ratio (18). The training set developed the model, while the validation set was used for internal validation. Initially, variables demonstrating statistical significance (*P* < 0.05) in univariate comparisons between normolipidemic and dyslipidemic subgroups were subjected to feature selection (nonzero coefficients) *via* least absolute shrinkage and selection operator (LASSO) regression. The λ.1se value was selected as the optimal threshold, emphasizing model simplicity while maintaining predictive stability (19, 20). Subsequently, variables retained by LASSO screening underwent multivariate logistic regression analysis. Finally, nomograms were plotted based on statistically significant risk factors from the multivariate logistic regression analysis. The final model was checked for multicollinearity by calculating the variance inflation factor (VIF). A sensitivity analysis was performed by means of the multiple imputation technique in order to determine whether the outcomes were influenced by missing data. Receiver operating characteristic (ROC) curves and the area under the curve (AUC) were employed to evaluate the predictive validity of the model. Calibration curves were utilized to determine the model’s accuracy, and the Hosmer-Lemeshow test was conducted to further assess goodness-of-fit. Additionally, decision curve analysis (DCA) and clinical impact curve (CIC) were applied to evaluate the model’s clinical utility. Standardized net benefit was calculated as the net benefit divided by the prevalence of dyslipidemia in the cohort, representing the proportion of additional true positives per 100 patients relative to the ‘treat-none’ strategy. *P* < 0.05 indicated a statistically significant difference. All statistical analyses were performed using R software (version 4.4.2).

## 3 Results

### 3.1 Clinical characteristics of the participants

This study included 913 participants in the analysis, of whom 580 (63.53%) were male and 333 (36.47%) were female. The median age was 12.26 years (IQR: 9.66–16.29 years). A total of 623 (68.24%) patients with dyslipidemia were classified according to their lipid profile as follows: 69 (11.08%) patients exhibited hypercholesterolemia, 200 (32.10%) had hypertriglyceridemia, 147 (23.60%) had mixed hyperlipidemia, 150 (24.08%) had low high-density lipoprotein hyperlipidemia, and 57 (9.15%) had hyper lipoprotein(a)emia. Elevated low-density lipoprotein cholesterol was observed in 107 patients out of a total of 913, including 81 patients with mixed hyperlipidemia and 26 patients with hypercholesterolemia.

Baseline characteristics stratified by dyslipidemic status are presented in **Table 1**. Age group (< 10 vs. ≥ 10 years), TC, triglyceride, HDL-C, LDL-C, ApoA-I, ApoB, the ApoA-I/ApoB ratio, lipoprotein(a), ALT, AST, GGT, Hcy, SOD, PLT, fibrinogen, UA and serum ceruloplasmin differed significantly between dyslipidemic and normolipidemic WD children (all *P*  <  0.05). Using stratified random sampling, participants were allocated to training (*n* = 641) and validation (*n* = 272) sets at a 7:3 ratio. For the clinical characteristics, there were no significant differences between the training and validation sets (all *P*  >  0.05; **Supplementary Table S2**).

**Table 1:** Clinical characteristics of the normolipidemic group and dyslipidemic group at the time of first hospital admission.

Characteristics	Overall (*n* = 913)	Normolipidemia group (*n* = 290)	Dyslipidemic group (*n* = 623)	*P* value
Sex				0.914
Male, *n* (%)	580 (63.53)	183 (63.10)	397 (63.72)	
Female, *n* (%)	333 (36.47)	107 (36.90)	226 (36.28)	
Age group				0.042
< 10 years old, *n* (%)	236 (25.85)	88 (30.34)	148 (23.76)	
≥ 10 years old, *n* (%)	677 (74.15)	202 (69.66)	475 (76.24)	
BMI (kg/m^2^)	19.42 (18.23, 20.72)	19.38 (18.12, 20.88)	19.46 (18.28, 20.67)	0.988
TC (mmol/L)	4.21 (3.42, 5.15)	3.80 (3.30, 4.42)	4.55 (3.52, 4.48)	< 0.001^*^
Triglyceride (mmol/L)	1.10 (0.73, 1.67)	0.80 (0.63, 1.03)	1.47 (0.87, 1.98)	< 0.001^*^
HDL-C (mmol/L)	1.25 (1.04, 1.49)	1.37 (1.17, 1.53)	1.17 (0.97, 1.46)	< 0.001^*^
LDL-C (mmol/L)	2.31 (1.77, 2.91)	1.97 (1.60, 2.46)	2.54 (1.89, 3.15)	< 0.001^*^
ApoA-I (g/L)	1.30 (1.14, 1.49)	1.36 (1.21, 1.50)	1.27 (1.14, 1.49)	< 0.001^*^
ApoB (g/L)	0.71 (0.54, 0.86)	0.61 (0.46, 0.71)	0.79 (0.61, 0.92)	< 0.001^*^
ApoA-I/ApoB ratio	1.92 (1.52, 2.45)	2.38 (1.90, 3.02)	1.76 (1.40, 2.20)	< 0.001^*^
Lipoprotein(a) (mg/L)	69.10 (29.00, 173.20)	51.40 (27.10, 93.60)	82.30 (30.65, 313.85)	< 0.001^*^
ALT (U/L)	44.00 (29.00, 82.00)	25.75 (16.00, 41.00)	55.00 (37.00, 107.55)	< 0.001^*^
AST (U/L)	37.00 (25.00, 55.00)	29.00 (21.00, 39.00)	42.00 (28.00, 66.25)	< 0.001^*^
GGT (U/L)	34.00 (21.00, 56.50)	26.00 (18.00, 40.00)	40.00 (23.00, 70.00)	< 0.001^*^
Albumin (g/L)	40.20 (37.70, 42.60)	40.10 (38.00, 42.00)	40.30 (37.60, 42.70)	0.549
TBIL (µmol/L)	10.41 (7.13, 15.95)	10.68 (7.59, 15.40)	10.30 (7.00, 16.15)	0.536
TBA (µmol/L)	5.20 (3.11, 9.80)	5.10 (3.30, 9.40)	5.20 (3.00, 9.87)	0.995
Hcy (μmol/L)	9.60 (7.15, 13.75)	8.85 (6.70, 12.70)	10.20 (7.40, 14.75)	< 0.001^*^
SOD (U/L)	196.00 (163.00, 214.00)	184.50 (148.00, 204.00)	200.00 (173.00, 223.00)	< 0.001^*^
PLT (× 10^9^/L)	224.00 (130.50, 286.50)	171.00 (116.00, 255.00)	242.00 (150.00, 297.00)	< 0.001^*^
Fibrinogen (g/L)	2.32 (2.10, 2.69)	2.25 (1.98, 2.61)	2.34 (2.13, 2.71)	< 0.001^*^
UA (µmol/L)	251.00 (185.50, 311.00)	231.50 (169.00, 290.00)	259.00 (194.50, 319.00)	< 0.001^*^
serum creatinine (µmol/L)	43.50 (33.85, 57.85)	43.70 (34.70, 58.60)	43.40 (33.55, 57.60)	0.517
BUN (mmol/L)	4.33 (3.61, 5.16)	4.40 (3.71, 5.24)	4.31 (3.58, 5.12)	0.242
serum ceruloplasmin (g/L)	0.06 (0.03, 0.10)	0.04 (0.02, 0.10)	0.06 (0.03, 0.10)	0.013
24-h urine copper (µg/24 h)	561.28 (324.93, 906.56)	540.83 (305.50, 919.61)	561.28 (343.31, 890.95)	0.590
serum copper (µmol/L)	3.42 (2.08, 5.37)	3.12 (1.98, 5.37)	3.42 (2.15, 5.37)	0.232

^*^ *P* < 0.05, dyslipidemic group compared with the normolipidemic group. BMI, body mass index; TC, total cholesterol; HDL-C, high-density lipoprotein-cholesterol; LDL-C, low-density lipoprotein cholesterol; ApoA-I, apolipoprotein A-I; ApoB, apolipoprotein B; ALT, alanine aminotransferase; AST, aspartate transaminase; GGT, gamma-glutamyl transpeptidase; TBIL, total bilirubin; TBA, total bile acid; Hcy, homocysteine; SOD, superoxide dismutase; PLT, platelet count; UA, uric acid; BUN, blood urea nitrogen. All continuous variables are presented as median (interquartile range, IQR).

### 3.2 Prediction of risk factors for dyslipidemia in children with WD

**Figure 2:**
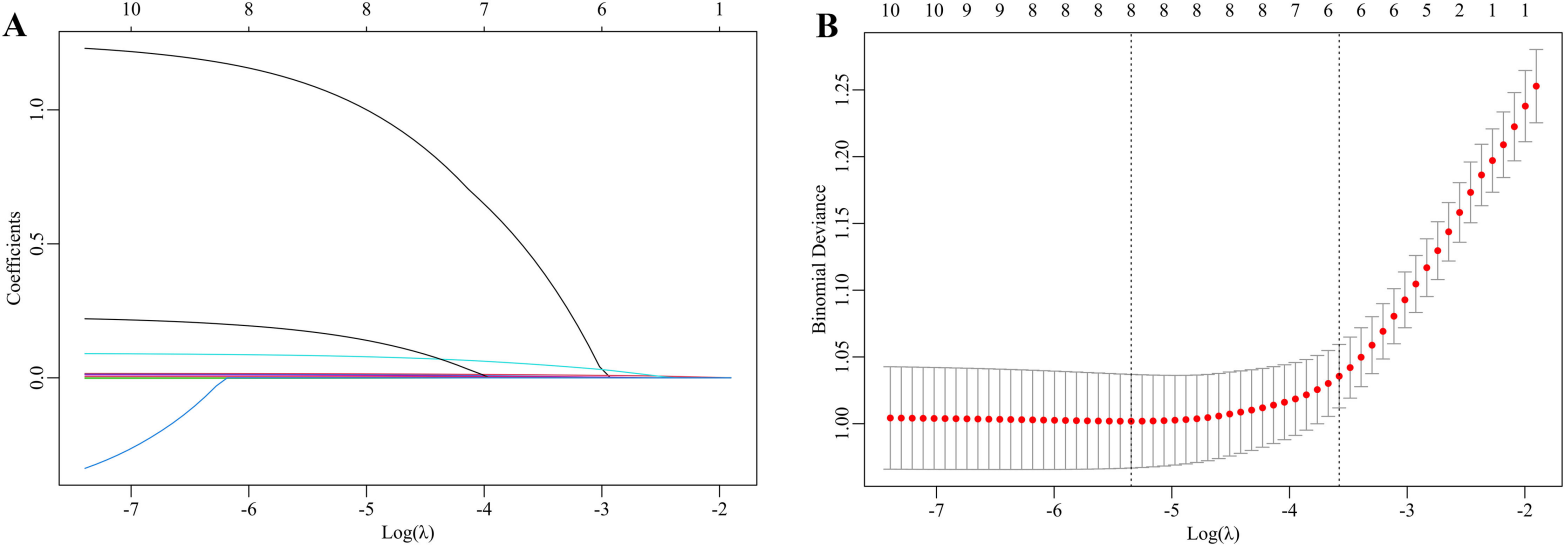
LASSO regression to select the risk factors for dyslipidemia in children with WD. **(A)** The coefficients of the LASSO regression model are presented across various logarithmic transformations of the lambda (λ) values. **(B)** The partial likelihood deviance is depicted for different log λ values within the LASSO framework. The left dashed line indicates the minimum λ, whereas the right dashed line represents the λ value at one standard error. The selection of the parameter lambda (λ) for the LASSO model was conducted using the one standard error criterion derived from a 10-fold cross-validation procedure. This process identified an optimal lambda value of 0.029. Consequently, six optimal features were ultimately selected on the basis of the LASSO coefficients.

Excluding the lipid metabolism indices, the 10 previously identified variables were further analyzed by LASSO regression. Within the training dataset, six variables with nonzero coefficients were identified through tenfold cross-validation, employing the one standard error criterion of lambda (λ) at a value of 0.029 (**Figures 2A, B**). These variables were age group (< 10 vs. ≥ 10 years), ALT, GGT, Hcy, SOD, and PLT.

Multivariate logistic regression models were subsequently constructed using six potential risk factors. In the training set, as shown in **Table 2**, ≥ 10 years old (OR = 3.065, 95% CI: 1.982–4.745), ALT (OR = 1.020, 95% CI: 1.014–1.027), GGT (OR = 1.010, 95% CI: 1.001–1.018), Hcy (OR = 1.094, 95% CI: 1.060–1.130), SOD (OR = 1.009, 95% CI: 1.005–1.013), and PLT (OR = 1.005, 95% CI: 1.002–1.007) were positively associated with dyslipidemic risk in children with WD. No significant collinearity was observed among variables (VIF range: 1.161–1.545).

**Table 2:** Multivariate logistic regression analysis in children with WD.

Variables	Odds ratio (95% CI)	*P* value
Age group		
< 10 years old	Ref	
≥ 10 years old	3.065 (1.982, 4.745)	< 0.001
ALT	1.020 (1.014, 1.027)	< 0.001
GGT	1.010 (1.001, 1.018)	0.026
Hcy	1.094 (1.060, 1.130)	< 0.001
SOD	1.009 (1.005, 1.013)	< 0.001
PLT	1.005 (1.002, 1.007)	< 0.001

ALT, alanine aminotransferase; GGT, gamma-glutamyl transpeptidase; Hcy, homocysteine; SOD, superoxide dismutase; PLT, platelet count.

The sensitivity analysis revealed a strong concordance between the complete-case and multiple imputation methodologies. Critical predictors, such as age group (< 10 vs. ≥ 10 years), ALT, GGT, Hcy, SOD, and PLT, exhibited nearly identical effect sizes and levels of statistical significance. These findings affirm the stability of the nomogram in the presence of missing data (**Supplementary Table S3**).

### 3.3 Nomogram for dyslipidemia risk prediction in children with WD

**Figure 3:**
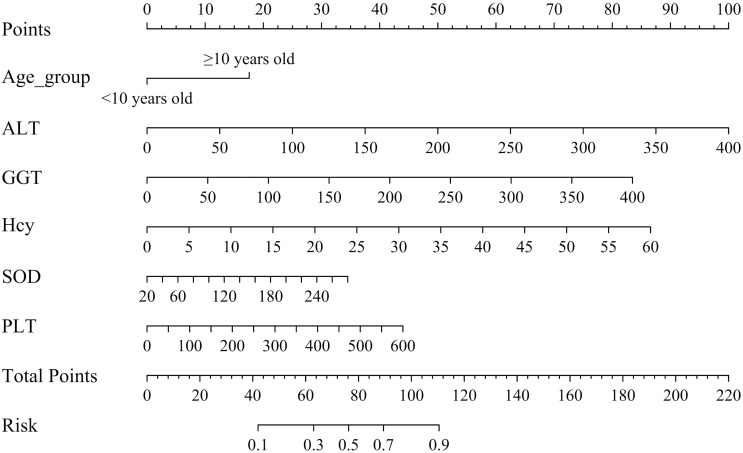
Nomogram for dyslipidemic risk prediction in children with WD. ALT, alanine aminotransferase; GGT, gamma-glutamyl transpeptidase; Hcy, homocysteine; SOD, superoxide dismutase; PLT, platelet count. Instructions for Utilizing the Nomogram: Each predictor is assigned a distinct score. To determine this score, the predictor’s position on the scale is identified, and a straight line is drawn to the corresponding scale above. The aggregate of these individual “points” constitutes the “total score,” which is then translated into the probability of a child with WD developing dyslipidemia.

As illustrated in **Figure 3**, the final nomogram was constructed utilizing six variables: age group (< 10 vs. ≥ 10 years), ALT, GGT, Hcy, SOD, and PLT. **Figure 3** serves as a predictive tool for assessing the likelihood of dyslipidemia in children diagnosed with WD. Each predictor is associated with a specific score. By locating its position on the scale and drawing a straight line to the scale above, the cumulative sum of each “point” yields the “total score,” which is subsequently converted into the probability of a child with WD developing dyslipidemia. For example, a 14-year-old participant with WD, presenting with an ALT level of 100 U/L, GGT level of 100 U/L, Hcy concentration of 15 μmol/L, SOD activity of 120 U/L, and PLT of 300 × 10^9^/L, exhibited a substantial likelihood of dyslipidemia, estimated at approximately 94.43% (95% CI: 88.80–97.32%).

### 3.4 Analysis of the discriminatory efficacy of the dyslipidemic model in children with WD

**Figure 4:**
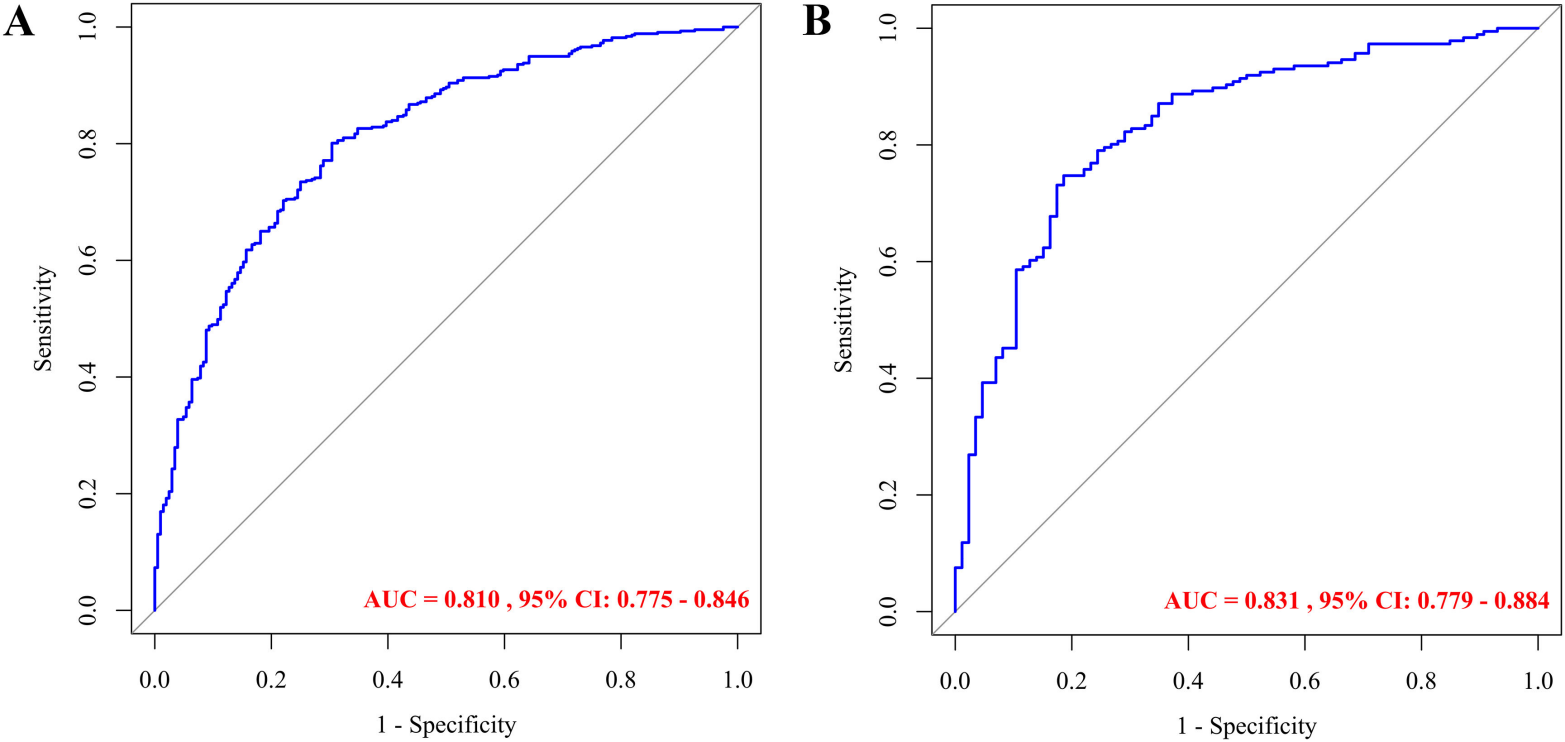
ROC curves for predicting dyslipidemia in children with WD: training set **(A)** and validation set **(B)**.

After constructing the model using the training dataset (*n* = 641), its predictive performance was evaluated on the validation dataset (*n* = 272). The model achieved an AUC of 0.810 (95% CI: 0.775–0.846) in the training set and 0.831 (95% CI: 0.779–0.884) in the validation set, indicating strong discriminatory power (**Figures 4A, B**). At the optimal cutoff value (training: 0.593; validation: 0.631), sensitivity and specificity were 0.801 and 0.696 in the training set, and 0.747 and 0.814 in the validation set, respectively. These results demonstrate high predictive accuracy of the nomogram.

### 3.5 Evaluation of the calibration performance of the dyslipidemic model in children with WD

**Figure 5:**
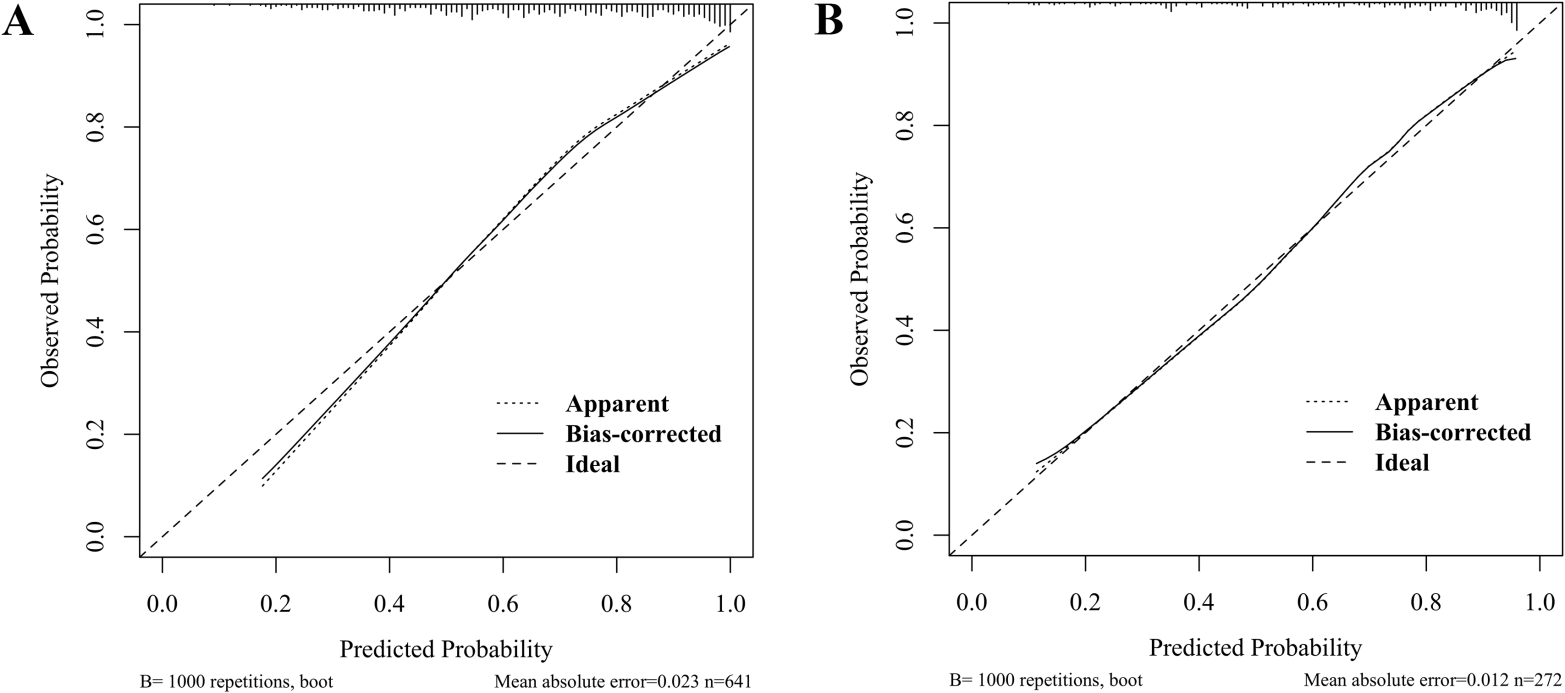
Calibration curves for predicting dyslipidemia in children with WD: training set **(A)** and validation set **(B)**.

Bootstrap-corrected calibration analysis demonstrated excellent agreement between the predicted and observed outcomes for dyslipidemia risks in pediatric WD, with mean absolute errors of 0.023 (training, **Figure 5A**) and 0.012 (validation, **Figure 5B**). The Hosmer-Lemeshow test yielded nonsignificant deviations (training: *P* = 0.474; validation: *P* = 0.280), indicating nonsignificant deviations. These results demonstrate the nomogram’s robustness in both the development and the internal validation cohorts.

### 3.6 Validation of the clinical utility of the dyslipidemic model in children with WD

**Figure 6:**
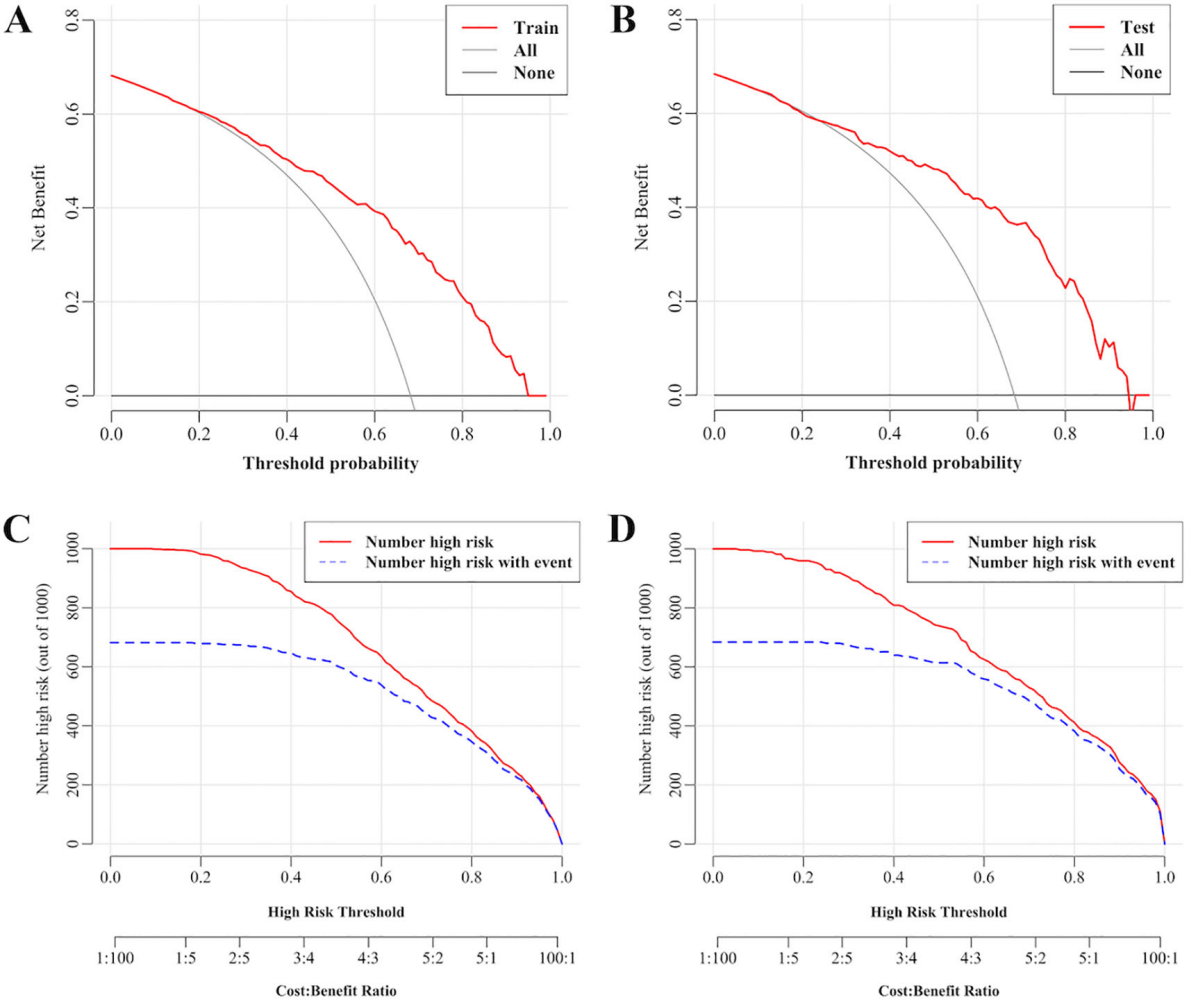
Decision curves and clinical impact curves for predicting dyslipidemia in children with WD. **(A)** Decision curves in the training set. **(B)** Decision curves in the validation set. **(C)** Clinical impact curves in the training set. **(D)** Clinical impact curves in the validation set. The x-axis represents threshold probabilities (i.e., the minimal risk probability justifying clinical intervention) and the y-axis quantifies the net clinical benefit of model-guided decision-making. The farther the decision curve is from the extreme curve, the greater the range of net benefit and the better the clinical utility. The x-axis integrates risk thresholds with cost–benefit ratios, whereas the y-axis enumerates high-risk cases per 1,000 individuals.

**Figure 6** validates the predictive model for dyslipidemia in children with WD through DCA and CIC. **Figure 6A** (training set) and **Figure 6B** (validation set) illustrate the decision curves, where the x-axis represents threshold probabilities (i.e., the minimal risk probability justifying clinical intervention) and the y-axis quantifies the net clinical benefit of model-guided decision-making. The model (red curves) demonstrated statistically superior net clinical benefits compared to the “treat-all” (gray dashed line) and “treat-none” (black dashed line) strategies across threshold ranges of 20–95% (training) and 25–95% (validation), underscoring its clinical utility for risk-stratified interventions. For instance, at a 50% threshold, the model achieved a net benefit of 48.5% (95% CI: 39.7–57.4%), sensitivity of 88.2% (95% CI: 83.1–92.8%), specificity of 62.8% (95% CI: 51.8–73.1%), and prevented 71 unnecessary interventions per 100 patients (standardized net benefit = 0.710) (**Supplementary Table S4**).

**Figures 6C, D** present the CICs. The x-axis integrates risk thresholds with cost–benefit ratios, whereas the y-axis quantifies high-risk cases per 1,000 individuals. A monotonic decline in both the predicted high-risk cases (red curve) and true event incidence (blue curve) was observed with increasing thresholds. These curves provide clinicians with a quantitative framework to optimize intervention thresholds by balancing early dyslipidemia prevention against overtreatment risks. Together, DCA and CIC confirm the model’s discriminative power and clinical applicability, establishing an evidence-based tool for personalized dyslipidemia management in pediatric WD.

## 4 Discussion

In this study, the prevalence of dyslipidemia among children with WD was determined to be 68.24%, indicating a relatively high occurrence. The incorporation of individuals aged 10 years and older, considered a high-risk group, alongside biomarkers including ALT, GGT, Hcy, SOD, and PLT—associated with hepatic injury, oxidative stress, and renal/metabolic dysfunction—establishes a multifaceted framework for dyslipidemia risk stratification in WD.

The nomogram model constructed in this study exhibited good discriminative power (AUC: 0.810 in the training set, 0.831 in the validation set) and calibration performance, indicating its accuracy. DCA and CIC further confirmed their clinical utility, offering a valuable tool for clinicians to make personalized decisions. The wide applicability of the model is evidenced by its clinical utility across threshold probabilities of 20%–95% (training) and 25%–95% (validation) in DCA. This broad effective range enhances its adaptability to diverse clinical scenarios, allowing clinicians to balance intervention intensity with individualized risk profiles. Thresholds < 20% may lead to overtreatment (specificity < 10%), while thresholds > 70% (e.g., 80%) result in missed high-risk cases (sensitivity drops to 48.4%) with diminishing net benefit (**Supplementary Table S4**). The threshold-dependent utility of the nomogram facilitates the development of tailored management strategies based on the clinical context. In high-resource settings, such as tertiary hospitals, lower thresholds (25–50%) are employed to prioritize sensitivity (88–98%), thereby enabling the early detection of dyslipidemia in children with concurrent risk factors. For instance, at a 25% threshold, clinicians might initiate follow-up appointments every three months, including lipid panels and liver function tests, for children who exceed this cutoff. Conversely, in resource-limited settings, such as primary care, higher thresholds (50–70%) are used to maximize specificity (63–84%), thereby reducing unnecessary referrals and concentrating on high-risk cases. For example, at a 50% threshold, children surpassing this probability are prioritized for specialist referral and pharmacotherapy if lifestyle modifications prove ineffective. Consequently, the constructed nomogram is promising as an effective noninvasive screening instrument for the early identification of dyslipidemia in pediatric WD patients.

The investigation of dyslipidemia in pediatric WD holds critical clinical significance. Dysregulation of hepatic lipid metabolism is an early feature of copper accumulation in WD (4, 21). This metabolic derange establishes a vicious cycle: copper-induced oxidative stress exacerbates hepatic steatosis and dyslipidemia, whereas lipid peroxidation products further amplify hepatocyte injury and fibrogenesis (5, 6). This pathophysiological interplay underscores the imperative for early detection, as delayed intervention permits progression to irreversible hepatic sequelae. Metabolic dysfunction-associated steatohepatopathy (MASLD) represents the most prevalent form of pediatric liver disease, impacting approximately 10% of the pediatric population (22, 23). The findings of the current study indicate that the incidence of dyslipidemia in pediatric patients with WD significantly exceeds that observed in cases of MASLD. A meta-analysis investigating the prevalence of dyslipidemia among Chinese individuals aged 2–18 years from 2015–2023 revealed an overall dyslipidemia prevalence of 19% in this population (24). Moreover, the United States Preventive Services Task Force reported that the prevalence of dyslipidemia among Americans aged 6–19 years was 19.2% from 2013–2016 (25). Notably, our findings revealed a 68.24% prevalence of dyslipidemia among children aged 3–18 years in this cohort, which was significantly higher than that reported in non-WD pediatric populations (24, 25). Furthermore, this study specifically identified hypertriglyceridemia and low high-density lipoprotein hyperlipidemia as the predominant dyslipidemic subtypes in pediatric WD patients, collectively constituting approximately 60% of the observed lipid abnormalities.

Current clinical guidelines lack WD-specific lipid management guidelines, particularly for pediatric populations. In this study, age was categorized based on diagnostic criteria, and a multifactorial analysis identified age ≥ 10 years as an independent risk factor for the development of dyslipidemia in pediatric patients with WD (OR = 3.065, 95% CI: 1.982–4.745). This finding may be indicative of the combined effects of physiological and metabolic changes occurring during adolescence. As a chronic condition characterized by copper accumulation, WD results in children aged ≥ 10 years experiencing a higher hepatic copper burden due to the prolonged duration of the disease, thereby contributing to more pronounced mitochondrial dysfunction and redox imbalance. Among the identified risk factors, ALT is a key indicator of liver damage and can disrupt lipid metabolism, leading to dyslipidemia. Despite new methods for liver assessment, ALT remains the primary global biomarker for liver injury (26). In pediatric WD patients, ALT levels are significantly elevated, with a median value of 44.00 U/L (IQR: 29.00–82.00), surpassing the normal range for their age (27–29). Our analysis revealed that ALT was an independent predictor of dyslipidemia, with each 10 U/L increase in risk by 20% (OR = 1.020, 95% CI: 1.014–1.027). This dose–dependent relationship aligns with previous studies demonstrating that every 10 U/L increase in ALT corresponds to an 11% increase in fatty liver risk among WD children (30). Elevated GGT, a marker of liver injury, is significantly linked to dyslipidemia in pediatric WD patients (median: 62 U/L vs. 38 U/L, *P* < 0.001). This elevation indicates potential cholangiocyte damage or cholestasis, affecting lipid absorption and increasing serum triglyceride and LDL levels (31).

GGT not only signals liver damage but also breaks down glutathione, producing pro-oxidants like cysteine-glycine, which enhance copper-induced lipid peroxidation (32). Research indicated that GGT levels are markedly increased in patients with WD who exhibit hepatic impairment (33). This oxidative stress, combined with copper overload, perpetuates dyslipidemia in WD.

Hcy is a metabolic intermediate product derived from methionine (34) that disrupts lipid homeostasis through pathways such as methionine metabolism issues (35), endoplasmic reticulum stress (36), and endothelial dysfunction (37). Mazi et al. reported that methionine metabolism was dysregulated in patients with WD (38). Clinical studies have demonstrated a positive correlation between elevated Hcy levels and hyperlipidemia (39). Additionally, Hcy modulates the metabolism of HDL-C and triglyceride (40) and is closely linked to dyslipidemia-associated comorbidities (41, 42). In this cohort study of 913 pediatric WD patients, the median Hcy level was 9.60 μmol/L (IQR: 7.15–13.75). Multivariate analysis revealed that Hcy was an independent risk factor for dyslipidemia, with an OR of 1.094 (95% CI: 1.060–1.130) per 1 μmol/L increase.

SOD, a key antioxidant enzyme, modulates oxidative stress and lipid metabolism (43). In WD, pathological copper accumulation directly induces mitochondrial ROS overproduction (44), which may trigger adaptive increases in SOD activity to counteract oxidative damage, particularly in early/asymptomatic stages as demonstrated by elevated SOD in presymptomatic pediatric WD carriers (Nagasaka et al.) (45). However, sustained oxidative stress could overwhelm this defense, leading to lipid peroxidation and dyslipidemia despite elevated SOD. This aligns with studies linking elevated SOD to atherogenic profiles (Canbay et al.) (46), suggesting its paradoxical role as both a compensatory responder and a marker of redox imbalance severity. Our study further demonstrated significantly higher SOD levels in dyslipidemic WD children than in normolipidemic WD children (median: 200.00 vs 184.50 U/L; *P* < 0.001), suggesting a compensatory antioxidant response to copper-induced redox imbalance. The combined inclusion of GGT, Hcy and SOD highlights oxidative stress as a pivotal mechanism in WD-associated dyslipidemia, which is consistent with established pathways of copper-mediated lipid peroxidation.

Platelet function and lipid metabolism are bidirectionally linked: their thrombotic-hemostatic activity depends on intrinsic lipid composition, whereas paracrine lipid release modulates inflammatory cell interactions (47, 48). Thrombocytopenia in WD patients primarily results from hypersplenism secondary to cirrhotic portal hypertension and diminished thrombopoietin production due to hepatic synthetic dysfunction. Interestingly, our study revealed elevated PLTs in pediatric WD patients with dyslipidemia, a paradoxical finding likely attributable to the exclusion of children with cirrhosis or hepatic failure in this cohort, thereby mitigating hypersplenism-related platelet consumption and preserving the PLT. However, alternative mechanisms such as subclinical inflammation (e.g., elevated CRP) or iron deficiency (e.g., reduced ferritin) could theoretically contribute to thrombocytosis. While these factors were not systematically assessed in our cohort, the mild ALT/AST elevations align more closely with copper-induced mitochondrial injury than overt inflammatory hepatitis. These findings underscore the complexity of platelet dynamics in WD. Future studies integrating inflammatory biomarkers, iron status, and platelet activation markers are warranted to delineate these multifactorial interactions. ALT, GGT, and PLT are integral components of routine biochemical and hematological panels in the majority of hospitals, characterized by low testing costs and swift turnaround times. At our institution, these biomarkers have been incorporated into comprehensive baseline metabolic panels owing to their significant clinical utility. While SOD and Hcy have not yet been integrated into WD-specific guidelines, it is anticipated that future prospective studies will provide evidence to support their routine application in clinical practice for patients with WD.

Although serum and urinary copper levels did not exhibit a correlation with lipid profiles, this paradox may be attributed to the discrepancy between systemic copper measurements and tissue-level copper toxicity. Notably, serum ceruloplasmin despite being elevated in dyslipidemic WD, was excluded by LASSO regression analysis. This exclusion could be due to serum ceruloplasmin’s context-dependent roles: it serves not only as a copper transporter but also as an acute-phase reactant that scavenges ROS. Its elevation might indicate a compensatory antioxidant response to chronic oxidative stress rather than a direct involvement in lipid metabolism. In our study, the selection of predictors was conducted with a critical approach, incorporating established non-invasive biomarkers for hepatic steatosis and fibrosis. This included key components from validated models: AST and PLT from the APRI; AST, ALT, and PLT from the FIB-4; and GGT, PLT, and albumin from the S-index. Additionally, BMI was included as a metabolic indicator. Furthermore, we integrated steatosis-specific indices, such as BMI, triglyceride, and GGT from the Fatty Liver Index (FLI), and ALT and AST from the Hepatic Steatosis Index (HSI).

### 4.1 Study strengths and limitations

This study represents one of the largest pediatric WD cohorts (*n* = 913) to date, a notable achievement given the rarity of WD and the challenges of recruiting pediatric populations for metabolic studies. Individuals aged ≥ 10 years (high-risk group), combined with biomarkers such as ALT, GGT, Hcy, SOD, and PLT—which are linked to hepatic injury, oxidative stress, and renal/metabolic dysfunction—provide a novel multifactorial framework for dyslipidemia risk stratification in WD.

However, several limitations warrant consideration. First, the retrospective design may introduce selection bias, despite rigorous statistical adjustments. Second, the single-center cohort limits generalizability, necessitating external validation in multiethnic populations. Third, dynamic changes in biomarkers during disease progression were not captured, which may affect longitudinal risk prediction. Fourth, while age ≥ 10 years emerged as a key non-biomarker risk factor, its dichotomization (< 10 vs. ≥ 10 years) was statistically derived rather than biologically validated, potentially oversimplifying puberty-associated metabolic transitions. Fifth, in order to preserve model simplicity and enhance clinical applicability, elastography parameters were excluded due to their limited availability in retrospective settings (less than 80% cohort coverage). Future prospective validations should incorporate these techniques. Finally, the clinical utility of the nomogram remains theoretical; prospective validation of risk-stratified interventions (e.g., antioxidant regimens for high-risk subgroups) is needed to confirm its impact on patient outcomes.

## 5 Conclusion

This study revealed that pediatric WD patients face a substantially elevated risk of dyslipidemia, with a prevalence of 68.24%, which is substantially greater than that of the general pediatric population. Focusing on the lack of WD-specific lipid management strategies, we developed and validated a novel nomogram integrating six clinically accessible predictors: age ≥ 10 years (high-risk group), ALT, GGT, Hcy, SOD, and PLT. The model demonstrated robust predictive accuracy (AUC: 0.810 in training, 0.831 in validation) and significant clinical utility across threshold probabilities (25–95%). Further validation in prospective cohorts and integration with dynamic biomarker monitoring are recommended to refine the clinical applicability of these findings.

## Data Availability

The raw data supporting the conclusions of this article will be made available by the authors, without undue reservation.
