# Supplementary material for: Development and validation of a nomogram model for prediction of dyslipidemia in children with Wilson disease: a retrospective analysis
Source: Front Endocrinol (Lausanne). 2025 Aug 21;16:1642083. doi: 10.3389/fendo.2025.1642083 (PMC12408305; doi:10.3389/fendo.2025.1642083)
Supplement: Supplementary file 1 [file Table1.docx]

Supplementary Material

## Supplementary Tables

**Supplementary Table 1. The number of missing data**

| **Variable** | **Amount of missing** | |
| --- | --- | --- |
|  | ***n*** | **%** |
| ApoA-I | 2 | 0.22 |
| ApoB | 2 | 0.22 |
| ApoA-I/ApoB ratio | 2 | 0.22 |
| Lipoprotein(a) | 26 | 2.85 |
| Hcy | 1 | 0.11 |
| SOD | 6 | 0.66 |
| PLT | 21 | 2.30 |

**Supplementary Table 2. Clinical characteristics of the training set and validation set at the time of first hospital admission**

| **Characteristics** | **Overall**  **(*n* = 913)** | **Training set**  **(*n* = 641)** | **Validation set**  **(*n* = 272)** | ***P* value** | |
| --- | --- | --- | --- | --- | --- |
| Dyslipidemia, *n* (%) | 623 (68.24) | 437 (68.12) | 186 (68.38) | 1.000 | |
| Sex | | | | 0.797 | |
| Male, *n* (%) | 580 (63.53) | 405 (63.18) | 175 (64.34) |  | |
| Female, *n* (%) | 333 (36.47) | 236 (36.82) | 97 (35.66) |  | |
| Age group | | | | 1.000 | |
| < 10 years old, *n* (%) | 236 (25.85) | 166 (25.90) | 70 (25.74) |  | |
| ≥ 10 years old, *n* (%) | 677 (74.15) | 475 (74.10) | 202 (74.26) |  | |
| BMI (kg/m^2^) | 19.42 (18.23, 20.72) | 19.46 (18.17, 20.73) | 19.36 (18.30, 20.67) | 0.996 | |
| TC (mmol/L) | 4.21 (3.42, 5.15) | 4.24 (3.43, 5.14) | 4.16 (3.38, 5.17) | 0.820 | |
| Triglyceride (mmol/L) | 1.10 (0.73, 1.67) | 1.11 (0.75, 1.65) | 1.09 (0.71, 1.70) | 0.368 | |
| HDL-C (mmol/L) | 1.25 (1.04, 1.49) | 1.25 (1.04, 1.50) | 1.25 (1.02, 1.49) | 0.455 | |
| LDL-C (mmol/L) | 2.31 (1.77, 2.91) | 2.29 (1.79, 2.90) | 2.37 (1.76, 2.98) | 0.626 | |
| ApoA-Ⅰ (g/L) | 1.30 (1.14, 1.49) | 1.31 (1.14, 1.50) | 1.30 (1.14, 1.47) | 0.942 | |
| ApoB (g/L) | 0.71 (0.54, 0.86) | 0.72 (0.54, 0.86) | 0.70 (0.56, 0.85) | 0.800 | |
| ApoA-Ⅰ/ApoB ratio | 1.92 (1.52, 2.45) | 1.92 (1.51, 2.46) | 1.89 (1.53, 2.42) | 0.730 | |
| Lipoprotein(a) (mg/L) | 69.10 (29.00, 173.20) | 66.60 (27.80, 176.20) | 72.30 (31.05, 168.80) | 0.338 | |
| ALT, U/L | 44.00 (29.00, 82.00) | 45.00 (30.00, 86.00) | 41.00 (28.10, 81.00) | 0.302 | |
| AST, U/L | 37.00 (25.00, 55.00) | 37.10 (25.00, 56.00) | 36.00 (24.05, 52.30) | 0.296 | |
| GGT, U/L | 34.00 (21.00, 56.50) | 36.00 (21.00, 59.00) | 31.00 (20.00, 53.00) | 0.137 | |
| Albumin (g/L) | 40.20 (37.70, 42.60) | 40.30 (37.70, 42.80) | 40.10 (38.05, 42.25) | 0.687 | |
| TBIL (µmol/L) | 10.41 (7.13, 15.95) | 10.49 (7.14, 15.90) | 10.11 (7.13, 15.90) | 0.763 | |
| TBA (µmol/L) | 5.20 (3.11, 9.80) | 5.21 (3.10, 10.18) | 5.06 (3.20, 9.30) | 0.818 | |
| Hcy (μmol/L) | 9.60 (7.15, 13.75) | 9.60 (7.10, 14.00) | 9.45 (7.25, 13.50) | 0.623 | |
| SOD (U/L) | 196.00 (163.00, 214.00) | 196.00 (163.00, 215.00) | 196.00 (162.50, 212.00) | 0.748 | |
| PLT (× 10^9^/L) | 224.00 (130.50, 286.50) | 217.00 (130.00, 284.00) | 235.50 (129.00, 290.00) | 0.642 | |
| Fibrinogen (g/L) | 2.32 (2.10, 2.69) | 2.34 (2.10, 2.69) | 2.30 (2.10, 2.71) | 0.593 | |
| UA (µmol/L) | 251.00 (185.50, 311.00) | 253.00 (184.00, 312.00) | 243.00 (189.00, 304.50) | 0.593 | |
| serum creatinine (µmol/L) | 43.50 (33.85, 57.85) | 43.70 (33.40, 57.80) | 43.10 (34.60, 57.75) | 0.910 | |
| BUN (mmol/L) | 4.33 (3.61, 5.16) | 4.32 (3.65, 5.14) | 4.40 (3.53, 5.17) | 0.899 | |
| serum ceruloplasmin (g/L) | 0.06 (0.03, 0.10) | 0.06 (0.03, 0.10) | 0.06 (0.03, 0.10) | 0.758 | |
| 24–h urine copper (µg/24 h) | 561.28 (324.93, 906.56) | 561.28 (334.90, 901.70) | 61.28 (314.36, 911.17) | 0.956 | |
| serum copper (µmol/L) | 3.42 (2.08, 5.37) | 3.42 (2.08, 5.37) | 3.42 (2.08, 5.37) | 0.947 |  |

*P* , validation set compared with the training set. Abbreviations: BMI, body mass index; TC, total cholesterol; HDL-C, high-density lipoprotein-cholesterol; LDL-C, low-density lipoprotein cholesterol; ApoA-Ⅰ, apolipoprotein A-I; ApoB, apolipoprotein B; ALT, alanine aminotransferase; AST, aspartate transaminase; GGT, gamma-glutamyl transpeptidase; TBIL, total bilirubin; TBA, total bile acid; Hcy, homocysteine; SOD, superoxide dismutase; PLT, platelet count; UA, uric acid; BUN, blood urea nitrogen. All continuous variables are presented as median (interquartile range, IQR).

**Supplementary Table 3. The results of sensitivity analysis of complete case (*n* = 884) and multiple imputation case (*n* = 913)**

| **Characteristics** | **Complete case (*n* = 884)** | |  | **Multiple imputation case (*n* = 913)** | |
| --- | --- | --- | --- | --- | --- |
|  | **OR (95% CI)** | ***P*** |  | **OR (95% CI)** | ***P*** |
| Age group |  |  |  |  |  |
| < 10 years old | Ref |  |  | Ref |  |
| ≥ 10 years old | 3.028 (1.968, 4.711) | < 0.001 |  | 3.065 (1.982, 4.745) | < 0.001 |
| ALT | 1.019 (1.013, 1.025) | < 0.001 |  | 1.020 (1.014, 1.027) | < 0.001 |
| GGT | 1.010 (1.002, 1.019) | 0.021 |  | 1.010 (1.001, 1.018) | 0.026 |
| Hcy | 1.097 (1.064, 1.137) | < 0.001 |  | 1.094 (1.060, 1.130) | < 0.001 |
| SOD | 1.009 (1.005, 1.014) | < 0.001 |  | 1.009 (1.005, 1.013) | < 0.001 |
| PLT | 1.004 (1.002, 1.007) | < 0.001 |  | 1.005 (1.002, 1.007) | < 0.001 |

Abbreviations: ALT, alanine aminotransferase; GGT, gamma-glutamyl transpeptidase; Hcy, homocysteine; SOD, superoxide dismutase; PLT, platelet count.

**Supplementary Table 4. Decision curve analysis results for nomograms with different thresholds in the validation set**

| **Risk Threshold** | **Sensitivity** | **Specificity** | **Net Benefit** | **Standardized Net Benefit** |
| --- | --- | --- | --- | --- |
| 0.20 | 0.995 (0.983–1.000) | 0.081 (0.025–0.142) | 0.608 (0.537–0.679) | 0.888 (0.855–0.917) |
| 0.25 | 0.984 (0.962–1.000) | 0.105 (0.039–0.173) | 0.578 (0.500–0.656) | 0.846 (0.801–0.884) |
| 0.30 | 0.973 (0.948–0.990) | 0.174 (0.092–0.259) | 0.554 (0.473–0.635) | 0.810 (0.756–0.855) |
| 0.35 | 0.973 (0.948–0.990) | 0.267 (0.177–0.371) | 0.541 (0.456–0.622) | 0.791 (0.729–0.843) |
| 0.40 | 0.930 (0.891–0.965) | 0.419 (0.310–0.522) | 0.513 (0.424–0.599) | 0.751 (0.671–0.817) |
| 0.45 | 0.914 (0.869–0.954) | 0.500 (0.392–0.607) | 0.496 (0.405–0.582) | 0.725 (0.636–0.798) |
| 0.50 | 0.882 (0.831–0.927) | 0.628 (0.518–0.731) | 0.485 (0.397–0.574) | 0.710 (0.619–0.791) |
| 0.55 | 0.828 (0.769–0.882) | 0.674 (0.579–0.776) | 0.440 (0.342–0.527) | 0.644 (0.539–0.729) |
| 0.60 | 0.774 (0.709–0.834) | 0.756 (0.665–0.852) | 0.414 (0.324–0.496) | 0.605 (0.503–0.699) |
| 0.65 | 0.715 (0.648–0.778) | 0.826 (0.744–0.903) | 0.387 (0.300–0.473) | 0.565 (0.452–0.664) |
| 0.70 | 0.624 (0.537–0.687) | 0.837 (0.751–0.911) | 0.306 (0.210–0.392) | 0.448 (0.312–0.552) |
| 0.75 | 0.554 (0.474–0.626) | 0.895 (0.827–0.964) | 0.279 (0.180–0.371) | 0.409 (0.274–0.523) |
| 0.80 | 0.484 (0.396–0.555) | 0.895 (0.827–0.964) | 0.199 (0.085–0.313) | 0.290 (0.127–0.441) |
| 0.85 | 0.387 (0.319–0.452) | 0.953 (0.908–0.989) | 0.181 (0.084–0.273) | 0.265 (0.132–0.389) |
| 0.90 | 0.290 (0.229–0.353) | 0.965 (0.921–1.000) | 0.099 (-0.038–0.211) | 0.145 (-0.06–0.296) |
| 0.95 | 0.172 (0.129–0.225) | 0.977 (0.941–1.000) | -0.022 (-0.248–0.134) | -0.032 (-0.374–0.200) |
